# Detection of Partially Structural Collapse Using Long-Term Small Displacement Data from Satellite Images

**DOI:** 10.3390/s22134964

**Published:** 2022-06-30

**Authors:** Alireza Entezami, Carlo De Michele, Ali Nadir Arslan, Bahareh Behkamal

**Affiliations:** 1 Department of Civil and Environmental Engineering, Politecnico di Milano, Piazza L. da Vinci 32, 20133 Milano, Italy; carlo.demichele@polimi.it (C.D.M.); bahareh.behkamal@polimi.it (B.B.); 2 Finnish Meteorological Institute (FMI), Erik Palménin Aukio 1, FI-00560 Helsinki, Finland; ali.nadir.arslan@fmi.fi

**Keywords:** structural health monitoring, collapse, displacement analysis, machine learning, synthetic aperture radar, bridges, TerraSAR-X

## Abstract

The development of satellite sensors and interferometry synthetic aperture radar (InSAR) technology has enabled the exploitation of their benefits for long-term structural health monitoring (SHM). However, some restrictions cause this process to provide a small number of images leading to the problem of small data for SAR-based SHM. Conversely, the major challenge of the long-term monitoring of civil structures pertains to variations in their inherent properties by environmental and/or operational variability. This article aims to propose new hybrid unsupervised learning methods for addressing these challenges. The methods in this work contain three main parts: (i) data augmentation by the Markov Chain Monte Carlo algorithm, (ii) feature normalization, and (iii) decision making via Mahalanobis-squared distance. The first method presented in this work develops an artificial neural network-based feature normalization by proposing an iterative hyperparameter selection of hidden neurons of the network. The second method is a novel unsupervised teacher–student learning by combining an undercomplete deep neural network and an overcomplete single-layer neural network. A small set of long-term displacement samples extracted from a few SAR images of TerraSAR-X is applied to validate the proposed methods. The results show that the methods can effectively deal with the major challenges in the SAR-based SHM applications.

## 1. Introduction

Bridges, high-rise buildings, dams, nuclear power plants, offshore structures, etc., are complex civil structures in every society. Apart from such modern systems, there are some ancient bridges, heritage buildings, and holy shrines that are taken into account as identities of a culture. Most of such structural systems have reached the end of their lifetime, owing to aging, old design and construction methodologies, material deterioration, environmental actions, excessive operational loads, etc. Under such cases, the occurrence of structural damage seriously threatens the safety and integrity of these civil structures. Natural hazards such as earthquakes, strong winds, floods, hurricanes, etc., significantly affect these structural systems and may cause partial or global collapses. In order to prevent such catastrophic events and increase structural safety, structural health monitoring (SHM) is a necessity for every society regardless of culture, geographical location, and economic development [1,2,3]. In general, this technology aims to evaluate the health and integrity of civil structures and alarm any abnormal conditions caused by damage occurrence at different levels (i.e., minor, moderate, heavy) and failure or collapses (i.e., partial or global).

Apart from the minor damage level, the moderate and heavy damage severities may cause partial and global collapses. In civil engineering, a collapse is a catastrophic event that causes the destruction of a civil structure due to natural hazards (e.g., earthquake, hurricane, strong wind, typhon, etc.). In a partial collapse, a small part of a civil structure collapses; however, the structure may preserve its whole stability. In this case, some important parts of the structure may remain safe, and one should repair or rehabilitate the damaged part of the structure for avoiding a global collapse. Economic and human (injury or death) losses of a partial collapse are significantly less than a global collapse. In a global collapse, however, the entire structure is destroyed during a catastrophic disaster. This kind of structural collapse is much more severe and dangerous compared to a partial collapse. The entire structure should be rebuilt after a global collapse, while it is possible to rehabilitate the damaged part of the structure in a partial collapse [4].

Generally, a classical SHM strategy relies on analyzing dynamic information (e.g., acceleration time series [5,6], modal data [7,8], both of them [9], etc.) acquired from contact-based sensors [10,11]. Despite valuable research studies on SHM via non-contact sensors [12,13], it is may be difficult to monitor large-scale civil structures and investigate pre-collapse in some critical civil structures such as dams and cross-sea bridges in large and wide areas. This problem becomes more difficult in long-term SHM projects, which may be implemented at every hour. An important challenge of the classical SHM methods is the lack of proper and sufficient information of civil structures from their initial stages or undamaged conditions. As there is no information about the initial states, the process of SHM is performed by available data, leading to a new challenge about the accuracy of SHM using incomplete data. To address these limitations, it is feasible to take advantage of synthetic aperture radar (SAR) satellite images, which make new technology in the context of SHM. The great merit of this technology is to provide an excellent opportunity for the health monitoring of civil structures [14,15,16]. The other advantages of the SAR-based SHM technology can be summarized as: (i) assessing large-scale civil structures in large and wide areas without sensor deployments, placements, and data acquisition systems, (ii) obtaining prior information (images) of civil structures, (iii) visualizing wide areas around structures, (iv) continuous and automated monitoring schemes, (v) preparing in place via an early warning monitoring system.

In this regard, Selvakumaran et al. [17] evaluated the bridge sour failure by analyzing 22 SAR images and eight target points on a bridge via the InSAR technique. Based on a substantial variation in displacements of one of the target points, they predicted an abnormal condition in the bridge. Qin et al. [18] analyzed time series displacements of 18 SAR images of a bridge structure and defined three target points on the bridge for SHM. Furthermore, Qin et al. [19] evaluated the health and performance of an arch bridge with a total of 96 SAR images of different satellites and orders by the multi-temporal DInSAR technique. Milillo et al. [20] monitored a dam in Italy by using a total of 201 SAR images of some satellites in a long-term SHM strategy from 2010 to 2015. The displacement samples from the center of the dam wall indicated variability in the data. Haung et al. [21] conducted a SHM strategy on a railway bridge in China by using 29 SAR images at six target points and analyzed the only displacements at these points obtained from a persistent scatterer technique along with their correlations with temperature fluctuations. Milillo et al. [22] assessed the pre-collapse potential of the Morandi Bridge in Italy by using a large number of SAR images of different satellites to conduct a long-term SHM and pre-collapse evaluation. By observing the only displacement samples at the vicinity of the collapsed area of the Morandi Bridge, they predicted the possibility of detecting the partial collapse.

In contrast to vibration-based SHM, which uses adequate large vibration data from wired or wireless sensors (i.e., 5956 modal samples [23,24] and 1,010,880,000 time series data [5,25]), the SAR-based SHM is usually implemented by *small data*. This means that although a SAR-based SHM scheme may be performed in a long-term manner (e.g., five years [20]), a small number of satellite images is often utilized to extract displacement samples. In other words, the term “small data” means that there may be insufficient or few data samples that can adversely affect the overall performances of machine (unsupervised) learners for decision making. Conversely, due to some restrictions (i.e., extremely large size of satellite images, the memory space limitations for collecting numerous images), the preparation of large displacement samples is not a trivial task. Therefore, SAR-based SHM is a process under small data. The other drawback of the classical SAR-based SHM is related to the direct analysis of small displacement samples. There is no doubt that civil structures are complex and expensive systems that play crucial rules in every society. In this case, any error in decision making/feature classification (i.e., false positive and false negative) may cause economic and human losses. Thus, the direct application of small data may not be an effective approach to SHM of critical civil structures, particularly in a continuous assessment scheme.

The other significant challenge in any long-term SHM is concerned with environmental/operational variability (EOV). Every civil engineering structure built in an open environment experiences ambient or environmental conditions. Depending upon the serviceability of the structure, it may be faced with different operational conditions. Prevalent environmental variations can be attributable to varying temperature and wind conditions, changing moisture and humidity levels [26,27,28]. Common operational conditions by considering the kind of serviceability are varying live-load conditions, varying mass loading, and changing operational and vehicle speeds. An important issue is that these conditions can change the inherent physical characteristics of the structure (i.e., mass and stiffness), materials, geometric and connectivity, leading to variations in vibration responses. In this regard, many field monitoring data demonstrate that measured responses of a civil structure not only pertain to the structural properties but are also highly susceptible to environmental and/or operational conditions. These phenomena in the structure are highly similar to circumstances that the structure suffers from damage. Because both damage and EOV conditions may cause similar changes in the structure, false positive/alarm and false negative/detection errors are prevalent in SHM [29]. In the context of SHM, a false positive error means that the structure of interest falls in its normal condition, but the methodology for SHM triggers an alarm regarding the emergence of damage. Because this issue causes redundant costs for structural condition assessment, it may be an economic loss. In contrast, a false negative error means that the structure may sustain damage, but the methodology proposed for SHM cannot accurately trigger the emergence of damage. Because this issue may threaten human health and may cause irreparable events, it is related to safety losses. Therefore, it is essential to remove any effect of the EOV conditions from monitoring data from either vibration features (e.g., modal frequencies) or displacement samples from SAR images.

Based on the aforementioned issues, the underlying goal of this research is to develop a classical SAR-based SHM technique by (i) augmenting the small set of displacement samples, (ii) removing or reducing the EOV conditions, and (iii) making decisions on the condition of the civil structure via machine learning algorithms. Accordingly, this article proposed two hybrid unsupervised learning methods whose main part is to augment small displacement samples extracted from satellite images by Markov Chain Monte Carlo (MCMC) and Hamiltonian Monte Carlo (HMC) samplers and remove the EOV conditions by feature normalization techniques. The first method, called MCMC-ANN-MSD, utilizes an artificial neural network (ANN) along with a new hyperparameter selection strategy to remove the EOV and reconstruct the augmented data (features) fed into the network, in which case the residual between the input and predicted data refers to normalized data or features (normalized augmented displacements). The second method, called MCMC-TSL-MSD, proposes a novel unsupervised teacher-–student learning via an undercomplete deep neural network (DNN) and an overcomplete single-layer neural network (SLNN). A new hyperparameter selection is also presented to find the number of neurons required for the hidden layers of the DNN. For the procedure of feature classification, this method applies the well-known Mahalanobis-squared distance (MSD). The main novelties of this research are summarized here. The first novelty is related to proposing two different multi-stage methods in terms of hybrid unsupervised learners for dealing with the three challenges in the classical SAR-based SHM. Each of these methods copes with all challenges in a single multi-stage framework. The second novelty pertains to the idea of data augmentation based on the MCMC and HMC sampler in an effort to extend small data for better visualization and analysis of variability conditions in long-term SHM. The third novelty relates to the problem of data normalization. Although the unsupervised auto-associative neural network was applied to SHM applications under variability conditions [30,31], the key novel element of this stage belongs to the proposed TSL method based on an undercomplete DNN (i.e., the teacher) and an overcomplete SLNN (i.e., the student); both of them are compatible with unsupervised learning. Finally, the fourth novelty is concerned with the problem of hyperparameter optimization. As clarified, despite the previous works of the auto-associative neural network, in this article, a new hyperparameter optimization is developed to determine the neuron sizes of the hidden layers of the auto-associative neural network. This development is innovative because the process of hyperparameter optimization is carried out on synthetic augmented data rather than real small data. Moreover, this process depends on the problem under study, that is, the mitigation of the environmental variations. Regarding the TSL method, another hyperparameter optimization strategy is proposed to determine the neuron sizes of the DNN and SLNN, of which this strategy is the other novelty. In order to prove the accuracy and high performance of the methods presented in this research, a small set of long-term displacement samples regarding the Tadcaster Bridge, Tadcaster, United Kingdom [17] is considered. The displacement samples were extracted from 45 satellite images of TerraSAR-X from March 2014 to November 2015 before its partial collapse on 19 November 2015. The results demonstrate that the proposed hybrid unsupervised learning methods effectively deal with the major challenges in the SAR-based SHM applications. Comparative analyses demonstrate that the proposed MCMC-TSL-MSD method is superior to the proposed MCMC-AAN-MSD.

## 2. Proposed Methods

### 2.1. Data Augmentation by Markov Chain Monte Carlo

The proposed methods aim at dealing with the problems of small data and EOV conditions. For the first problem, both methods exploit a data augmentation strategy based on the MCMC and HMC algorithms to augment small displacement samples. It is necessary to clarify that although one can utilize different standard and simple data generation algorithms for data augmentation, this article takes advantage of the HMC technique owing to its more robust and rigorous sample evaluation. To put it another way, after drawing new data points, this technique evaluates whether the augmented or generated data points are compatible with a reasonable set of random realizations from the target distribution. In this regard, the Gelman–Rubin convergence statistic makes sure whether the chains have converged to the target distribution. If the statistic does not meet its standard, it is essential to draw more Monte Carlo samples. Using the augmented data samples, these are applied to visualize the EOV. In statistics and the theory of probability, MCMC is a statistical technique for sampling data points by characterizing a probability distribution function. This technique simulates random data samples without any knowledge about its mathematical characteristics [32]. The word “Monte Carlo” makes sense of estimating the properties of a distribution function based on the examination of random data points. Moreover, the word “Markov Chain” denotes a sequential way for making random samples so that new samples do not rely upon any data before the previous one [33].

Conversely, the HMC sampler is taken into account as a gradient-based MCMC technique that intends to make data points from a target probability density function, i.e., the multivariate Gaussian distribution in this study, of **d** [34]. Recently, Entezami et al. [35] exploited this technique to develop a probabilistic threshold estimator for damage localization using a small set of outputs. In this research, one attempts to take advantage of this technique for data augmentation. The HMC sampler is represented by a logarithmic function of the target distribution, the gradient of this function, and a momentum vector **λ**. With these descriptions, it is necessary to define a Hamiltonian function *H*(**d**,**Ω**) through Hamiltonian dynamics in the following form:

(1) H(δ,λ)=U(δ)+V(λ)

where **δ** ∈ **d**; *U*(**δ**) denotes the logarithmic function of the probability of interest and *V*(**λ**) = ½ **λ**^T^**T**^−1^**λ,** where **T** is a positive definite, diagonal, and symmetric matrix. Accordingly, *V*(**λ**) is defined as minus the logarithmic probability density of the zero-mean Gaussian distribution with the covariance matrix **T** [34]. As such, Hamiltonian dynamics run on **δ** and **λ** to derive Hamiltonian expressions that intend to determine how the vectors **δ** and ϕ change over time, *t*, as:

(2) $$\frac{d\delta_{i}}{dt}=\frac{\partial H}{\partial \lambda_{k}}=\frac{\partial V\left(\lambda \right)}{\partial \lambda_{k}}$$

(3) $$\frac{d\lambda_{i}}{dt}=\frac{\partial H}{\partial \delta_{k}}=\frac{\partial U\left(\delta \right)}{\partial \delta_{k}}$$

where *k* = 1,2,…,*p*. According to Equations (2) and (3), and the initial quantities of **d_0_** and momentum **λ**_0_ at time *t*_0_, one can simulate **d** and **λ** at *t* = *t*_0_ + Δ*t*, for which Δ*t* is the step size through the leapfrog technique [34]. Accordingly, the main objective is to forecast **d** under pre-defined chain (*C*) and sampling (*N*) numbers. Hence, the HMC approach is run to draw *N* samples of **d** from the target probability distribution $D\in {\mathbb{R}}^{p\times N}$, *C* chains under an iterative strategy (i.e., *i* = 1,…,*C* and *j* = 0,…,*N* − 1 for sampling $D_{j+1}^{\left(i\right)}$) and the acceptance probability criterion from the Gelman–Rubin convergence statistic [36]. If the convergence statistic holds, the simulated parameter at the (*j* + 1)th iteration is fixed; otherwise, the simulated parameter of the previous, *j*th, iteration should be selected. Once the *C* sets of **D** have been obtained, the mean value of C sets is utilized as the final multivariate dataset. The same HMC sampling process should be run for any new small dataset. In the context of machine learning, the process of decision making is based on defining training and test datasets. Accordingly, the augmented data matrix **D** is divided into two parts **X** $\in {\mathbb{R}}^{p\times n}$ and **Z** $\in {\mathbb{R}}^{p\times m}$, where *n* < *N* and *m* < *N.*

### 2.2. Feature Normalization of MCMC-ANN-MSD

**Figure 1:**
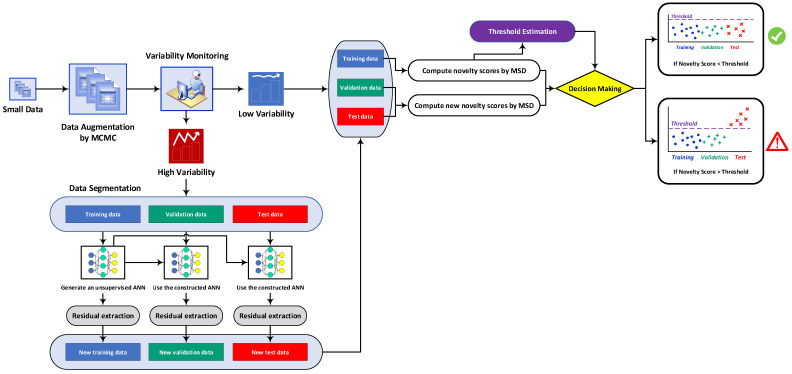
Flowchart of the proposed multi-stage unsupervised learning method.

This section intends to describe the process of feature normalization by the auto-associative neural network, which is the second part of the proposed MCMC-ANN-MSD technique (i.e., the first hybrid approach). Before the procedures of feature normalization and classification, a concise discussion on the proposed hybrid method is carried out to facilitate the understanding of its implementation and performance. Apart from the first part of data augmentation (Section 2.1), in the context of SHM, the feature normalization or data normalization is a technique for removing the EOV conditions from the main features [37] Chapter 12. Feature classification is intended to initially determine novelty indices/scores concerning the training and test data points, where the training data depend only on the undamaged state of the structure, and they discriminate the novelty indices of the damaged state from the normal one by using a decision threshold [7,24]. In the first proposed hybrid unsupervised learning method MCMC-ANN-MSD, the process of feature normalization is based on an unsupervised ANN under the auto-associative neural network configuration. For feature classification, the proposed method utilizes the well-known MSD novelty detector (see Section 2.4) due to its simplicity and computational efficiency. In this case, the MSD value acts as a novelty score for decision making. This novelty detector does not perform well under strong EOV conditions [24,31]. However, since this issue is dealt with by the proposed feature normalization method, it is possible to apply the MSD as an effective novelty detector. For convenience, Figure 1 shows the main flowchart of the proposed multi-stage unsupervised learning method.

In Figure 1, the process begins by applying a set of small displacements obtained from the SAR images. Using the MCMC algorithm, this set is augmented to prepare a large dataset of displacement samples. Since the EOV conditions are of paramount importance to SHM, a variability monitoring or evaluation step is considered to find the level of the variability on the augmented data. For this step, the simplest way is to apply graphical tools such as box plot or variance plot. Based on the level of variability (i.e., small or large), it is possible to decide whether to apply feature normalization or continue without it. This option is added as an alternative when the civil engineer recognizes that the EOV conditions are not serious. On this basis, the augmented data are divided into the training, validation, and test sets, and these sets are applied to the MSD for decision making.

In the first step of SHM, the only data of the undamaged condition of the structure are available. Hence, such data are initially decomposed into the training and validation sets. Using the training samples and the MSD values from these samples, one can define a decision threshold for the final feature classification. In this regard, since the validation data are associated with the undamaged structural state, one expects that their novelty indices/scores (i.e., MSD values) fall below the threshold limit similar to the novelty scores of the training samples. By obtaining an augmented test set regarding the current (unknown) state of the structure, its novelty score is compared with the threshold. If it passes the decision threshold, the method should trigger the alarm of damage occurrence; if not, it has a novelty score similar to the training and validation samples; that is, a novelty score or distance value below the threshold, in the sense of the undamaged state of the structure. If the civil engineer detects that the level of variability is high, one needs to apply the process of feature normalization. On this basis, new features or normalized augmented data can be determined that are applied to the MSD based on the training, validation, and test sets.

#### 2.2.1. Unsupervised Auto-Associative Neural Network

In artificial intelligence, ANNs are computational models inspired by biological neural networks for learning a process. Generally, they are used to approximate functions that are generally unknown. The main objective of an unsupervised ANN is to map an input into a desired output. For this objective, an ANN consists of some layers and neurons. The neuron in each layer includes weights, biases, summation and transfer functions. This is a simple simulation of a biological cell body [38]. In most cases, the network architecture, the number of layers and neurons, and the summation and transfer functions are considered before the training process. In such a case, the main task is to estimate or adjust the weights and biases.

The auto-associative neural network is one of the tried-and-tested unsupervised ANNs that mainly intends to develop an approximation of mapping between inputs and outputs using a backpropagation network with feed-forward connections, linear or sigmoid transfer functions, and back-propagation training algorithms [39]. The network learns a mapping from given inputs to desired output values by adjusting internal weights to minimize a least-square error objective function. The auto-associative neural network utilizes a feed-forward architecture consisting of an input layer, three hidden layers (i.e., mapping, bottleneck, and de-mapping layers), and an output layer. The data samples fed into the network are mounted onto the input layer. The output layer produces a filtered version of the inputs so that it has the same dimension with the input layer. An important note about the network topology is that the bottleneck layer, which is of paramount importance to the functionality of the auto-associative neural network, should contain a smaller number of neurons compared to the mapping and de-mapping layers and their sum [39]. Unsupervised learning behavior and the ability to eliminate outliers, noise, and variability conditions are the great merits of the auto-associative neural network [31,39].

More precisely, the auto-associative neural network is viewed as a serial combination of two single-hidden layer networks. The input, mapping, and bottleneck layers represent the nonlinear function *G* aiming at projecting the feature samples (inputs) to a lower dimension space. This mapping is expressed as follows:

(4) $$b_{i}=G_{i}\left(x\right),\;\;\;i=1,2,\ldots ,l_{b}$$

where *b_i_* denotes the output of the *i*th bottleneck node, *l_b_* represents the number of nodes (neurons) of the bottleneck layer, and **x** = [x_1_…x*_p_*]^T^ is the vector of the *p*-dimensional feature samples or inputs from the augmented (training) data **X**. The bottleneck, de-mapping, and output layers make the second network with another nonlinear function *H* that reproduces an approximation of the inputs from the factors at the output of the bottleneck layer in the following form:

(5) $${\hat{x}}_{j}=H_{j}\left(b\right),\;\;j=1,2,\ldots ,l_{m}$$

Using all feature vectors from **X** = [x_1_…x*_n_*], where each vector consists of *p* samples, the auto-associative neural network reconstructs the input data in the output layer $\hat{X}$ = [$\hat{x}$_1_…$\hat{x}$*_n_*]. Hence, one can remove the effects of the EOV by determining the residual between the input and output sets as follows:

(6) $$E_{x}=\left|X-\hat{X}\right|$$

Having considered the test matrix **Z**, the process of feature normalization for these sets is performed by using the learned ANN considering its main configuration (i.e., the number of hidden layers and neurons as well as the estimated weight and bias values) and reconstructing the new test samples in the output layer. Finally, its residual matrix is extracted as the normalized augmented data:

(7) $$E_{z}=\left|Z-\hat{Z}\right|$$

**Figure 2:**
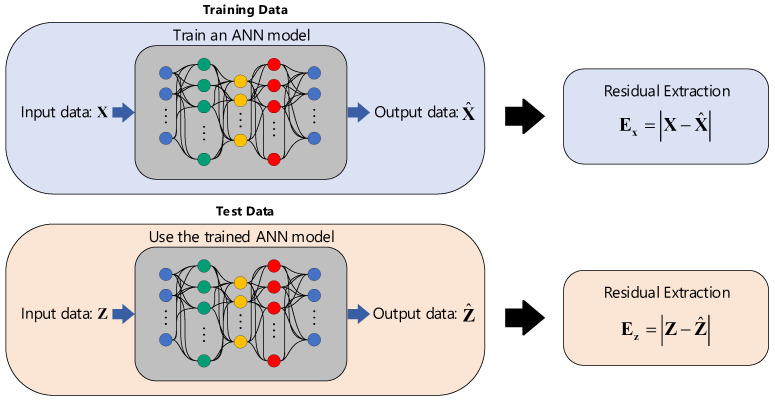
The graphical representation of the unsupervised feature normalization by ANN.

Accordingly, Figure 2 displays a graphical representation concerning the ANN-based feature normalization by using the training and test datasets. As can be seen, the residual extraction refers to the process of removing the EOV conditions.

#### 2.2.2. ANN Hyperparameter Selection

The hyperparameters are unknown elements of a parametric machine learning algorithm that directly affects its general performance [31,40]. In this case, an inappropriate choice of these parameters causes an inaccurate learning process. By contrast, model parameters are also other unknown parameters of a machine learning algorithm that is usually obtained in an automated manner during the learning process. Although the determination of these parameters depends on the algorithm of interest, the hyperparameters should be selected priorly before the learning process. For example, the number of hidden layers and neurons in ANN applications or the number of clusters in partition-based clustering algorithms are some important hyperparameters of unsupervised learning algorithms. Moreover, the weight and bias values of neurons estimated in the learning process of an ANN are some examples of the model parameters. Due to applying an ANN to feature normalization, it is important to determine its hyperparameters.

One of the advantages of the auto-associative neural network is that the number of hidden layers is known, which corresponds to three (i.e., the mapping, bottleneck, and de-mapping) layers. Hence, the only hyperparameter is the number of neurons of these hidden layers. In most cases, some general criteria such as mean-square-error between the inputs and outputs of the ANN are used to determine the hyperparameter. Nonetheless, these criteria may not be applicable to a specific problem such as SHM. Since the false positive and false negative errors directly relate to the economic and safety issues, in this report, an effective iterative algorithm is proposed to obtain the number of neurons of the hidden layers with a focus on decreasing the impacts of the EOV cases. The main idea behind this method is to use relatively large sample neurons and to check the variance of the outputs or novelty scores of the novelty detector—the MSD in this study. The fundamental principle is that a robust feature normalization method effectively enables the novelty detector to produce smooth scores with a small variance rate [7]. Algorithm 1 lists the main steps of the proposed hyperparameter selection for the number of neurons of the hidden layers. In this algorithm, $l_{m_{0}}$ and $l_{b_{0}}$ are the sample neuron numbers of the mapping as well as de-mapping layers and bottleneck layer, respectively. As Krammer’s recommendation [39,41], the number of neurons of the first (mapping) and third (de-mapping) layers should be larger than the bottleneck layer. Moreover, in order to avoid any overfitting problem, the following equations should be held [41]:

(8) $$l_{b}<2l_{m}<\frac{p\left(n-l_{b}\right)}{p+l_{b}+1}$$

(9) $$2l_{m}<n$$

Since the learning process of an auto-associative neural network is compatible with the unsupervised learning, the augmented training samples in **X** should be used to determine the hyperparameter and to learn the ANN. On this basis, the trained ANN is applied to the augmented test data for feature normalization.
**Algorithm 1.** The main steps of the hyperparameter selection of the number of neurons of the hidden layers of an auto-associative neural network.**Inputs**: Input data **X**, $l_{m_{0}},$ and $l_{b_{0}}$, where $l_{m_{0}\;}$>$\;l_{b_{0}}$**For** *i* = 1: $l_{m_{0}}$ **Do**            **For** *j* = 1: $l_{b_{0}}$ **Do**            1.   Learn a network through the *i*^th^ and *j*^th^ number of neurons of the mapping/de-mapping and bottleneck layers.
            2.   Determine the output of the network, that is $\hat{X}$.            3.   Calculate the residual $E_{x}\;=\;|X-\hat{X}|$.
            4.   Apply the residual matrix to the MSD equation as a new training dataset
            5.   Determine distance values using all samples in **E_x_**.
            6.   Calculate the variance of the calculated distance quantities regarding the *i*^th^ and *j*^th^ numbers.
            7.   Save the calculate variance amount in a matrix, where the *i*^th^ row and *j*^th^ column belong to this amount.
            
**End**
**End**8.   Select the smallest quantity of the stored variances from Step 7.9.   Check the occurrence of overfitting by Equations (7) and (8). In the case of an occurring overfitting problem, go to Step 8 and choose the next smallest variance amount to make sure that the overfitting problem does not occur.10.  Select the numbers associated with the stored matrix row and column with the smallest variance amount that pertains to the optimal number of neurons of the mapping/de-mapping (*l_m_*) and bottleneck (*l_b_*) layers, respectively.**Outputs**: The optimal number of neurons *l_m_* and *l_b_*

### 2.3. Feature Normalization of MCMC-TSL-MSD

**Figure 3:**
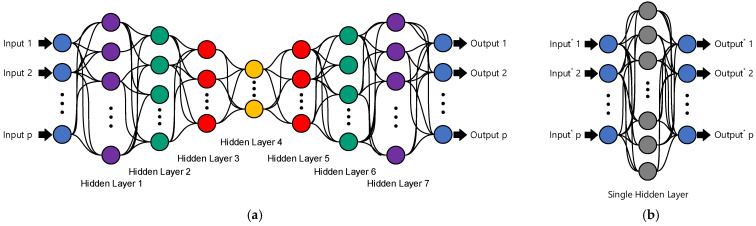
(**a**) Deep feedforward neural network with undercomplete configuration; (**b**) single-layer neural network with overcomplete configuration.

The other approach to providing robust decision making for SAR-based SHM strategy relies on feature normalization under a teacher–student learning method. This technique comes from the main idea of the teacher–student learning with two differences. First, the proposed method is based on unsupervised learning. Second, it develops by combining a deep neural network (DNN) as a teacher (see Figure 3a) and a single-layer neural network (SLNN) with an overcomplete configuration, which acts as a student (see Figure 3b). The main objective is first to learn the DNN by using the augmented data **X**. The DNN of interest is a seven-layer undercomplete feedforward configuration, as shown in Figure 3, which is able to reconstruct the inputs at the output layer. On this basis, similar to the previous method, the residuals between the inputs and outputs are extracted to use as a new input and to feed into the SLNN. Hence, the process of the residual extraction is repeated in the SLNN to extract the new residual set from the difference between the previous residual matrix from the DNN and the outputs in the output layer of the SLNN.

**Figure 4:**
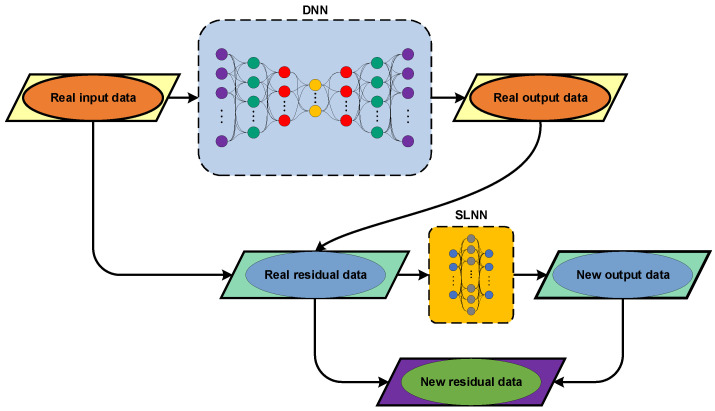
The flowchart of the proposed TSL.

Having considered the test matrix **Z**, the trained DNN (teacher) and SLNN (student) are applied to feed Z into these networks and to repeat the same residual extraction procedures to extract the final residual set of the test matrix in the output layer of the trained SLNN. The graphical representation of the proposed TSL method is shown in Figure 4. In this figure, the real input and output data refer to the augmented training matrix **X** and the reconstructed input data at the output layer, which is defined here as $\bar{X}$. Hence, the residual between the real input and output data can be extracted as $E_{x}=X-\bar{X}$. As described, this residual matrix is used as the new input data and feeds into the SLNN to reconstruct it as ${\bar{E}}_{x}$. Finally, the residual data obtained from the difference between the new input data $E_{x}$ and the new output data ${\bar{E}}_{x}$ are extracted as ${\overset{=}{E}}_{x}=E_{x}-{\bar{E}}_{x}$. Once the residual matrices related to the training and test samples have been obtained, those are applied to the MSD for computing novelty scores.

#### 2.3.1. TSL Hyperparameter Selection

**Figure 5:**
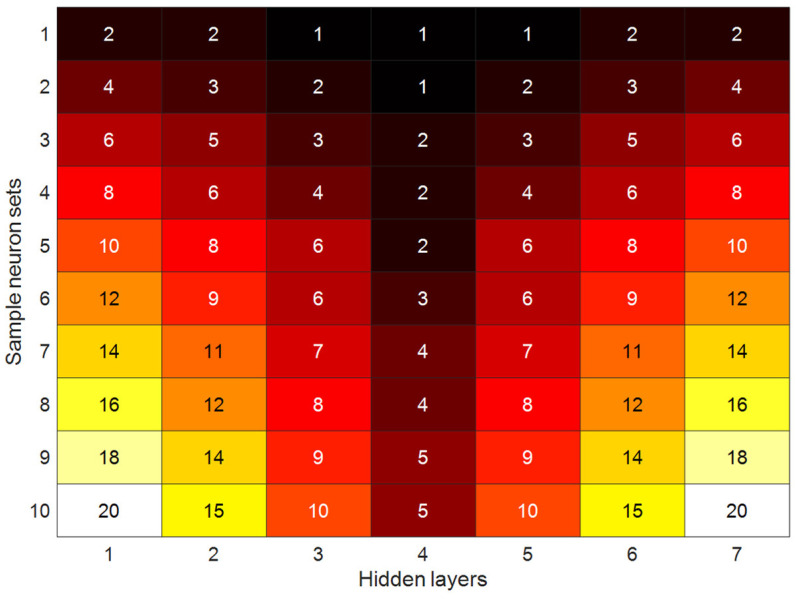
Sample neurons for hidden layers of the DNN.

Since the proposed TSL method consists of two ANNs, it is necessary to determine the number of neurons of the seven hidden layers of the DNN and one single layer of the SLNN. Inspired by Entezami et al. [42], who developed a hyperparameter selection algorithm for deep autoencoders, another approach is proposed here by using the Akaike information criterion (AIC) under sample neuron sizes depicted in Figure 5. Accordingly, it only suffices to run the TSL algorithm and compute the sum of residual samples. Let us define *h*_1_–*h*_7_ as the neuron sizes of the seven hidden layers. Hence, the AIC is expressed as follows:

(10) $$\mathrm{AIC}=\ln\left(e\right)+\frac{2N_{w}}{N}$$

where *N* = *p* × *n*, *N_w_* =$\;\left(p+h_{4}+1\right)\left(\overset{7}{\underset{i=1}{\sum }}h_{i}\right)+\left(p+h_{4}\right)$, and *e* is a scalar value called an average sum of squared errors defined as *e* = *E*/2*N*, where:

(11) $$E={\sum_{i=1}^{n}\sum_{j=1}^{p}\left(e_{x_{j}}-{\bar{e}}_{x_{j}}\right)}_{i}^{2}$$

where $e_{x}{}_{j}$ and ${\bar{e}}_{x}{}_{j}$ are the *j*^th^ row samples of the new residual matrix $E_{x}$ (i.e., the new input data fed into the SLNN) and the DNN new output ${\bar{E}}_{x}$. By examining the sample neurons of the seven hidden layers presented in Figure 5, the sample (row) with the minimum AIC value is chosen as the optimal neuron sizes.

Once the optimal number of neurons of the seven hidden layers of the DNN have been determined, the neurons of the single layer of the SLNN need to be select. An important note is that a small neuron size may not have influential effects on the performance of the learning process. Moreover, the choice of a large number increases the probability of overfitting. Conversely, the neuron size of interest should be larger than the input samples (*p*) to make an overcomplete SLNN configuration. For these reasons, the sum of the optimal neuron sizes of the hidden layers of the DNN larger than *p* is incorporated as the optimal neuron size of the single layer *h_s_*; that is, *h_s_* =$\;\overset{7}{\underset{i=1}{\sum }}h_{i}$.

### 2.4. Novelty Detection by MSD

Once the low-dimensional displacement samples have been augmented and then normalized, they are applied to the MSD for novelty detection. This process is implemented in the baseline and monitoring stages. Using the matrix **X** for low variability conditions (i.e., the feature normalization is not necessary) or **E_x_** for high variability conditions, the main objective is to estimate a mean vector $m_{x}\in {\mathbb{R}}^{p}$ and covariance matrix $\Sigma_{x}\in {\mathbb{R}}^{p\times p}$. Since the training matrix has the multivariate Gaussian distribution, one can ensure that the utilization of sample mean and covariance estimators provides accurate outputs (i.e., the mean vector and covariance matrix). Using these components, the MSD is derived as:

(12) $$d_{{\mathrm{MSD}}_{i}}={\left(x_{i}-m_{x}\right)}^{T}\Sigma_{x}^{-1}\left(x_{i}-m_{x}\right)$$

or

(13) $$d_{{\mathrm{MSD}}_{i}}={\left(e_{x_{i}}-m_{x}\right)}^{T}\Sigma_{x}^{-1}\left(e_{x_{i}}-m_{x}\right)$$

where *i* = 1,2,…,*n* and $e_{x}{}_{i}$ is a vector of the residual matrix **E_x_**. Accordingly, it can be determined that *N* distances $d_{MSD_{1}},\ldots ,d_{MSD_{n}}$. These *n* distances are also considered to estimate a decision threshold. Based on the augmented test samples in **Z** or the residual matrix **E_z_**, each of their vectors is applied to the MSD equation by incorporating the estimated mean vector and covariance matrix from the augmented training matrix. For simplicity, one assumes that the validation matrix or its residual matrix is merged into the test set. Hence, the MSD can be rewritten as follows:

(14) $${\bar{d}}_{{\mathrm{MSD}}_{i}}={\left(z_{j}-m_{x}\right)}^{T}\Sigma_{x}^{-1}\left(z_{j}-m_{x}\right)$$

or

(15) $${\bar{d}}_{{\mathrm{MSD}}_{i}}={\left(e_{z_{j}}-m_{x}\right)}^{T}\Sigma_{x}^{-1}\left(e_{z_{j}}-m_{x}\right)$$

where *j* = 1,2,…,*m*. As such, *m* distance values ${\bar{d}}_{MSD_{1}},\ldots ,{\bar{d}}_{MSD_{m}}$ regarding the monitoring phase can be obtained. By collecting the *n* and *m* distance values of the baseline and monitoring stages into the distance vector **d***_MSD_* = [$d_{MSD_{1}},\ldots ,d_{MSD_{n}},{\bar{d}}_{MSD_{1}},\ldots ,{\bar{d}}_{MSD_{m}}$]$\;\in {\mathbb{R}}^{N}$, the process of decision making is implemented by comparing each of the distance quantities in **d***_MSD_* with the estimated threshold boundary from the baseline phase. Similar to the previous method, it is expected that the first and second *N* distance values regarding the training and validation sets are under the decision threshold, implying the undamaged state of the structure. For the third *N* distance quantities, one can reach the mentioned three decision-making conditions:

- If each of the distance values exceeds the threshold boundary, one should trigger the emergence of damage
- If the distance values are under the threshold boundary, one should ensure the safe condition of the structure.

## 3. Application: The Tadcaster Bridge

To prove the correctness and high performance of the proposed hybrid methods, this article exploits the small set of displacement samples belonging to the Tadcaster Bridge, Tadcaster, North Yorkshire, United Kingdom [17]. The main reasons for applying this case study can be summarized as: (i) this is a realistic case of a partial collapse of a full-scale civil structure, (ii) a long-term monitoring strategy was considered to analyze satellite images and extract the displacement samples, and (iii) an appropriate target point selection was incorporated, such as the placement of vibration-based sensors, which enables us to evaluate the displacement changes throughout the bridge.

### 3.1. A brief Description of the Bridge

**Figure 6:**
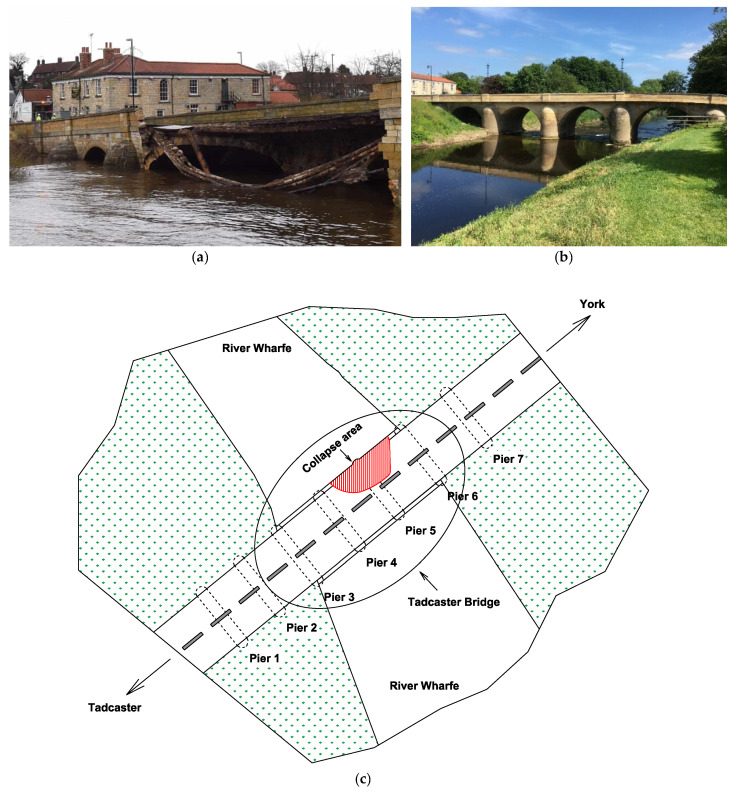
(**a**) The Tadcaster Bridge and its collapsed area [17]; (**b**) the bridge after reconstruction of the damaged area; (**c**) the plan view, pier labels, and collapse area.

The Tadcaster Bridge is a historical nine-arch masonry bridge over the River Wharfe in Tadcaster, United Kingdom. The road bridge is dated from around 1700. It is the main way that connects the two sides of the town and one of two road crossings in the town, the other being the bridge for the A64 bypass. The total length of the bridge is approximately 100 m with a width of 10 m, carrying a single lane of vehicular traffic in each direction and a pedestrian walkway on each side. Before the partial collapse, the present bridge comprised two structures of different dates, built side by side to expand the width of the original structure. Documentary evidence shows that it was built from 1698 to 1699, replacing an earlier bridge on the same site that had been swept away by a flood. Flooding events in recent years prior to the collapse meant that the bridge was inspected by divers to detect movement of the riverbed that may have resulted in scour. The Tadcaster Bridge partially collapsed on 29 December 2015 after flooding that followed Storm Eva and reopened on 3 February 2017 after a major reconstruction of the damaged area. Figure 6a,b illustrates the images of the damaged area of the Tadcaster Bridge and its reconstruction. Figure 6c also shows the plan view of the bridge and its collapse (damaged) area.

**Figure 7:**
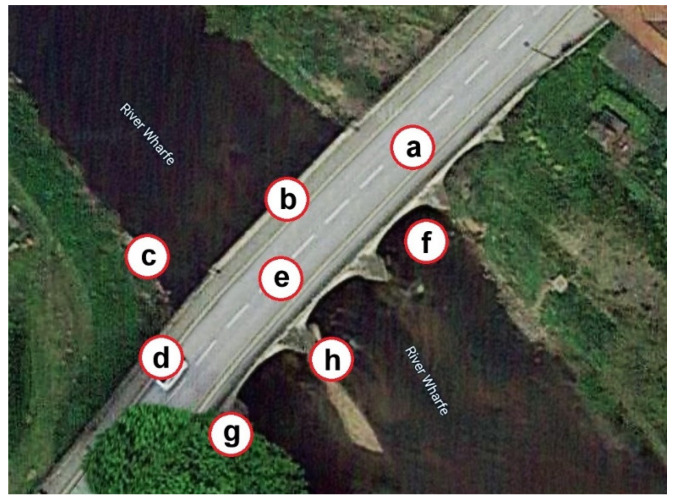
The eight points of displacement samples as designated by “a”, “b”, “c”, “d”, “e”, “f”, “g”, and “h” (i.e., point “b” is the location of the partial collapse) [17].

**Figure 8:**
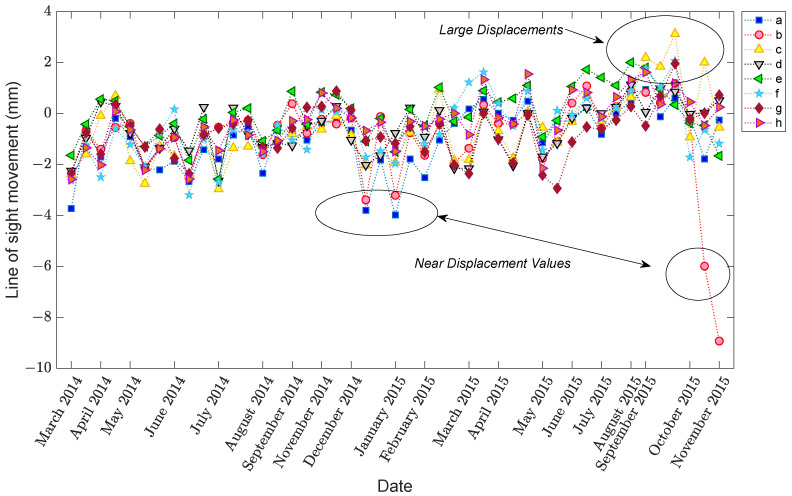
Displacement samples of the eight points [17].

To analyze the deformation behavior in the period preceding collapse, 45 TerraSAR-X Stripmap mode images (3 × 3 m ground resolution) taken prior to the collapse in the period from 9 March 2014 to 26 November 2015 were considered and studied. The final acquisition in November was the last image available prior to the bridge collapse on 29 December 2015. These image acquisitions were taken at 11-day intervals where possible. Based on the DInSAR methodology and Small Baseline Subset (SBAS) technique, a small set of displacement samples at eight points, designated by “a”, “b”, “c”, “d”, “e”, “g”, “h”, and “f”, as shown in Figure 7, was extracted. Conversely, Figure 8 shows the variations in the displacements of these eight points. From this figure, it is observed that the small set of displacement samples in a long-term monitoring scheme includes randomly unpredictable variations. Apart from the last two displacement samples regarding images #44 and #45, there is large displacement deviation that may pertain to the EOV conditions. Moreover, the amounts of displacement samples are positive and negative, which means that the direct analysis of changes in displacements for decision making is slightly questionable. Although the Tadcaster Bridge falls into the moderate-span bridge type, the mentioned drawback for larger bridges is much more serious. Therefore, it would be risky for an expensive and important civil structure that the only visual observation of the displacement variations is the main step of a SAR-based SHM strategy.

### 3.2. Data Augmentation and EOV Evaluation

**Figure 9:**
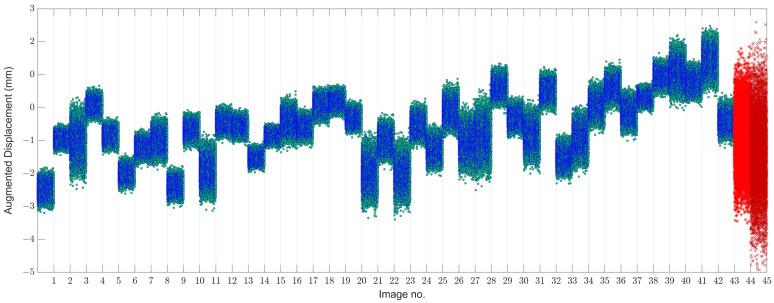
Augmented displacement samples based on the MCMC and HMC sampler by using the 100 sample points.

To further assess the variability in small displacement data, the MCMC based on the HMC sampler is applied to augment the data and provide augmented displacement samples. For this process, the numbers of extended samples *N* and chains are set as 100 and 10, respectively, in which case each displacement sample converts to a set with 100 samples leading to the augmented displacement data of the size 8 × 4500, as shown in Figure 9.

**Figure 10:**
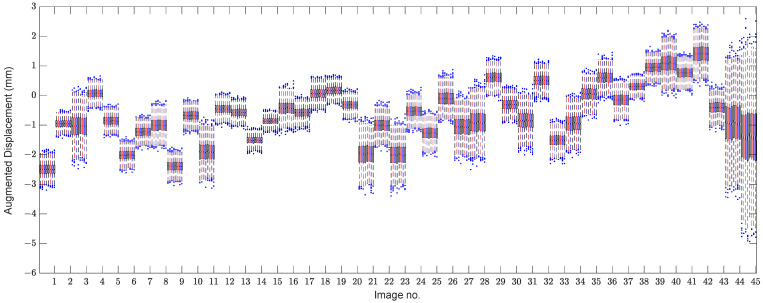
Box plot of the augmented displacement samples.

Compared to Figure 8, Figure 9 takes some advantages that enable us to clearly observe the variations in the structure due to the EOV or other variability conditions. For further evaluation, Figure 10 depicts the box plot of the augmented displacement data. In both figures, the theory of data augmentation assists us in better observing the variations in structural responses and behavior. As can be seen in Figure 10, the augmented samples of some images include large variances, particularly regarding the last two images. In this regard, one can observe that the last image has the largest variance. This indicates how the aforementioned theory interprets variations in real data appropriately.

To evaluate the performances of the proposed methods for the collapse prediction or for early damage detection, the augmented displacement samples are divided into the training, validation, and test data points. For this aim, the first 41 satellite images of the Tadcaster Bridge are used to make the training data. On this basis, the training matrix $X\in {\mathbb{R}}^{8\times 4100}$. Having considered that the last two images pertain to the damaged condition, each of the validation and test (damaged) data include 200 augmented samples. The combination of the validation and test samples is considered as the final test data $Z\in {\mathbb{R}}^{8\times 400}$.

### 3.3. Verification of the Proposed MCMC-ANN-MSD Method

**Figure 11:**
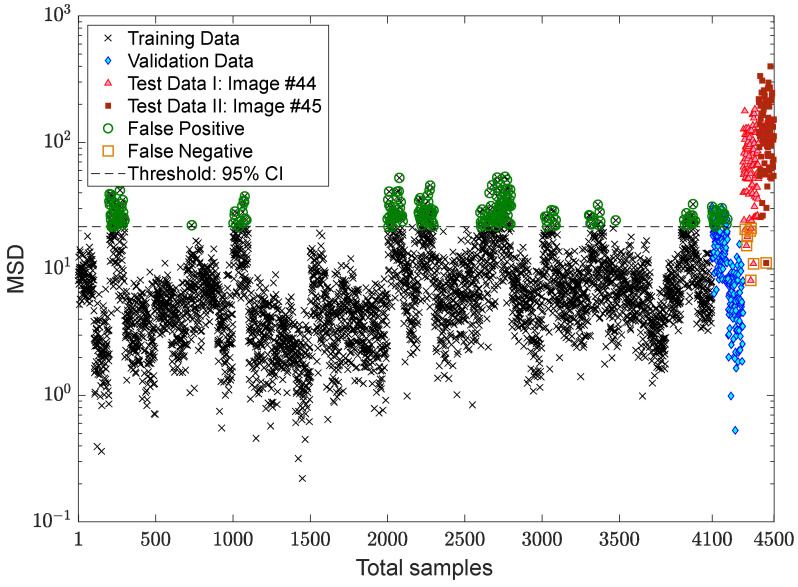
Damage detection of the Tadcaster Bridge without feature normalization.

In this section, one initially attempts to show the effect of the EOV condition or any variability source in the displacement samples on the decision-making process. Hence, the first result relies on the distance computation by the MSD method without any feature normalization as shown in Figure 11. To make a decision, the standard confidence interval based on a 5% significance level of the distances of the training data points is computed to obtain a decision threshold limit, which is detectable as a dashed line in the mentioned figure. As can be observed, the variations in the augmented displacements are also available in the distance values. In other words, due to large variations in the MSD values of the samples 1–4100, it is difficult to make a confident decision. In the context of SHM, the outputs or novelty scores of the normal condition should be as smooth as much as possible [7]. The same result can be seen in the validation samples. Although due to the augmentation of the small data, the distance values of the last two images differ from the training and validation samples, most of them are over the threshold, and some distance values of the undamaged condition (the samples 1–4300) have large MSD amounts.

**Figure 12:**
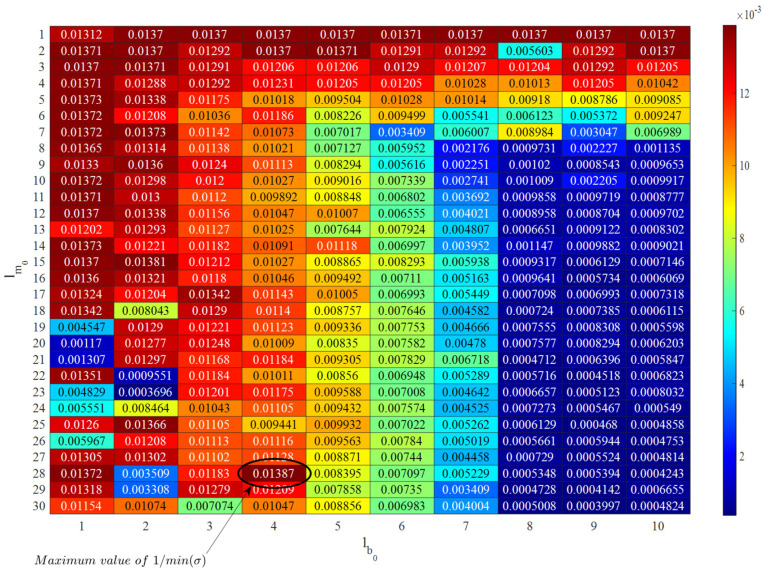
Hyperparameter selection of the auto-associative neural network.

To demonstrate the positive effect of feature normalization on removing or reducing the EOV conditions, an auto-associative neural network is learned by the training data. Based on the proposed hyperparameter optimization approach, the sample numbers of the neurons for the mapping/de-mapping and bottleneck layers correspond to 30 and 10, respectively. Accordingly, Figure 12 displays the inverse of the variance values obtained from Algorithm 1 for finding optimal neurons of these layers, which are equal to 28 and 4, respectively. The inverse of the variance values is used to better observe the hyperparameter selection result. For learning the auto-associative neural network, the Levenberg–Marquardt backpropagation is applied to perform estimates of the model parameters under 1000 epochs.

**Figure 13:**
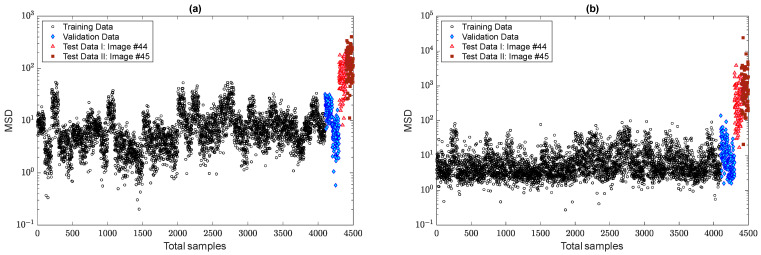
Evaluation of the effect of the ANN-based feature normalization: (**a**) MCMC-MSD; (**b**) MCMC-ANN-MSD.

In the next step, the residual matrices of the training and test samples are computed to apply to the MSD method for feature classification. For a comparison without the alarming threshold, Figure 13a,b indicates the MSD values using and without using the ANN-based feature normalization. As can be observed in samples 1–4100, the variability in the MSD quantities reduced significantly, and the outputs (novelty scores) of the training samples became smoother. The other improvement is on the small variations regarding the validation data, the samples 4101–4300 (i.e., the green marker in the right plot of Figure 13). Finally, the last improvement is the increase in the MSD values of samples 4301–4500 concerning the last two images. This means that the implementation of the feature normalization not only reduces the effect of the EOV but also increases the damage detectability of the proposed method.

**Figure 14:**
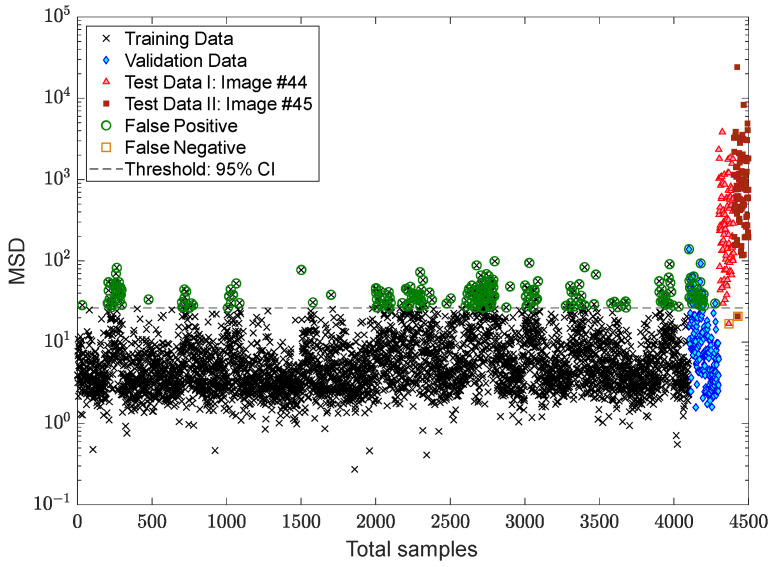
Damage detection of the Tadcaster Bridge using MCMC-ANN-MSD and the CLT-based threshold estimator.

**Figure 15:**
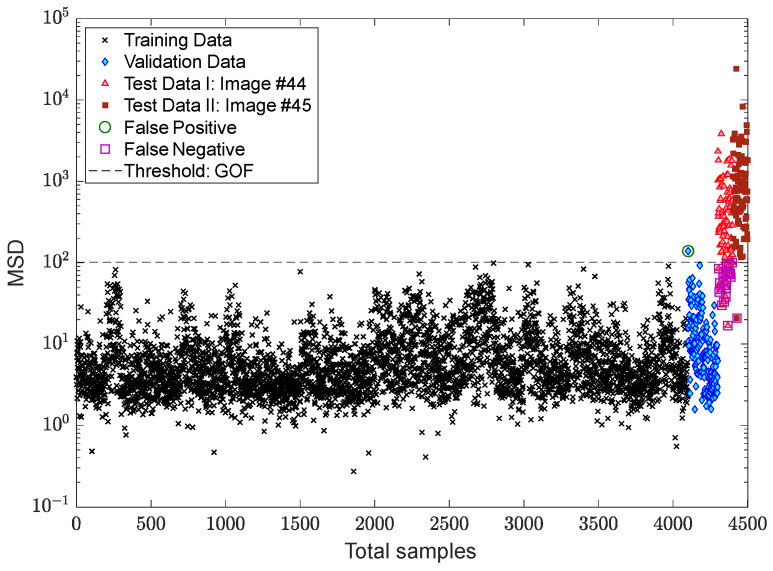
Damage detection of the Tadcaster Bridge using the GOF-based threshold estimator.

After succeeding in dealing with the problem of the EOV conditions, the process of decision making is followed by applying two kinds of threshold limit. The first threshold is based on the 95% confidence interval of the MSD values of the training samples, while the second one relates to the EVT-based threshold estimator proposed by Sarmadi et al. [23]. The results of early damage detection are shown in Figure 14 and Figure 15. Despite properly reducing the EOV, it is observed in Figure 14 that some false alarms are available in the training and validation samples. This most likely makes sense that the standard confidence interval under the central limit theory is not suitable for this issue or that the removal of the EOV should be performed better. Using the same novelty scores, Figure 15 demonstrates that there is no false alarm in the training sample, and only one point regarding a validation sample exceeds the threshold. Nonetheless, the rate of false negative regarding image #44 increases due to determining a larger threshold limit based on the EVT. However, the occurrence of this error also depends on the use of displacement samples of the undamaged area of the bridge, that is, the points “a”, “c”–“f”. Accordingly, it can be seen in Figure 15 that all novelty scores of the last image are over the threshold.

### 3.4. Verification of the Proposed MCMC-TSL-MSD Method

**Figure 16:**
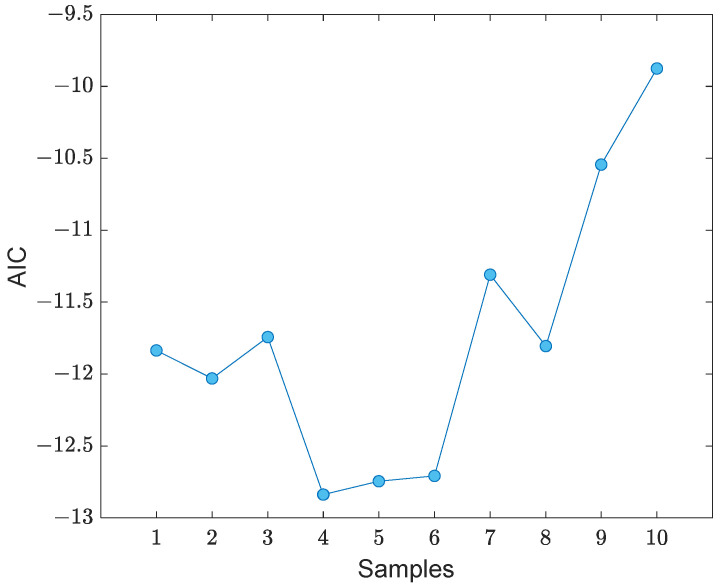
Selection of the optimal number of neurons of the seven hidden layers of the DNN.

For early damage detection by the proposed TSL method, it is first necessary to select the optimal number of neurons concerning the seven hidden layers of the DNN. Based on the hyperparameter selection approach presented in Section 2.3.1, the iterative algorithm is performed by using the sample neurons as illustrated in Figure 5 and by running the TSL process. Accordingly, it can be determined to be 10 AIC values for all neuron samples as shown in Figure 16. As can be observed, the fourth, fifth, and sixth sample sets of the neuron sizes are smaller than the other sets. Hence, the average of these three sets is chosen as the optimal neurons, which correspond to 10, 8, 6, 3, 6, 8, 10. In this case, the optimal neuron of the single layer of the SLNN is equal to *h_s_* = 51.

**Figure 17:**
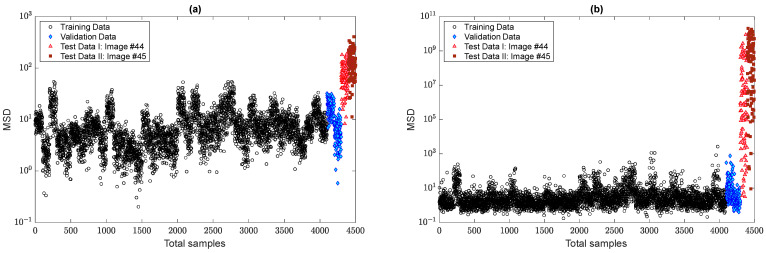
Evaluation of the effect of the TSL-based feature normalization: (**a**) MCMC-MSD; (**b**) MCMC-TSL-MSD.

To demonstrate the positive effect of the proposed TSL method in feature normalization, Figure 17 compares the novelty scores of the MCMC-MSD, without any feature normalization, with the proposed MCMC-TSL-MSD method. As expected, it is seen that the novelty scores of the training and validation samples in Figure 17b are much smoother with a smaller rate of variance compared to the corresponding samples in Figure 17a. This indicates the positive effect of the proposed TSL method in removing the EOV conditions. Another important observation is that the proposed method significantly increases the damage detectability, for which the MSD values of test samples 4301–4500 in Figure 17b are highly larger than the corresponding values in Figure 17a. Please compare the scales of the vertical axes of these plots. A graphical comparison between Figure 17b and Figure 13b discloses that the proposed TSL algorithm is better than the proposed ANN-based feature normalization under the three-hidden-layer auto-associative neural network in terms of smoother novelty scores and higher damage detectability.

**Figure 18:**
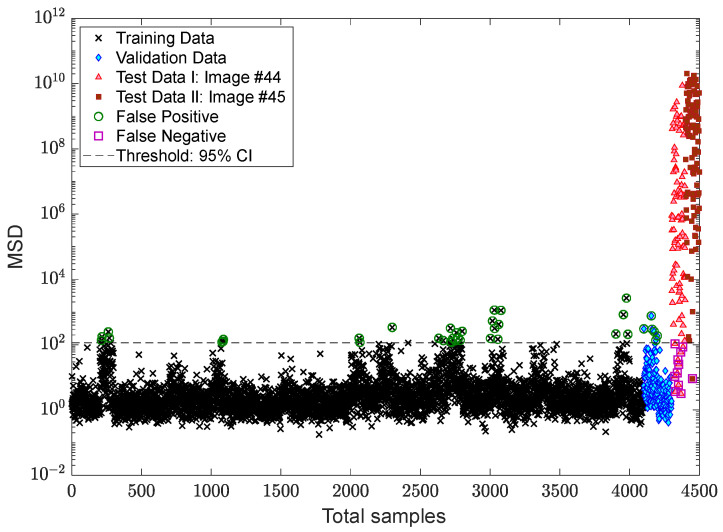
Damage detection of the Tadcaster Bridge using MCMC-TSL-MSD and the CLT-based threshold estimator.

For more precise outputs of damage detection, Figure 18 shows the novelty scores obtained from the proposed MCMC-TSL-MSD method, which are compared with a decision threshold gained by the 95% confidence interval of the novelty scores of the training samples. As can be seen, the use of the proposed TSL approach significantly reduces the rates of false positive/alarm and false negative/detection errors even with the classical CLT-based threshold estimator. The comparisons of this figure with Figure 14 and Figure 15 demonstrate that the current proposed method provides simpler and more confident decision making with one kind of the threshold estimator. This method neither gives a high rate of false positive similar to Figure 14 nor a high rate of false negative, such as Figure 15. Hence, due to the benefit of the proposed TSL method in significantly reducing the EOV conditions, providing smoother novelty scores, and increasing damage detectability, it is not necessary to apply rigorous threshold estimators such as EVT-based techniques. 

## 4. Conclusions

This work intended to develop the classical SAR-based SHM technique for a long-term monitoring scheme via small data. For this reason, new multi-stage unsupervised learning methods are proposed to detect early damage such as partial collapses under EOV conditions. The main parts of these methods consisted of data augmentation via the MCMC and HMC sampler, ANN-based feature normalization, and feature classification via the well-known MSD technique. On this basis, the first and second parts of the proposed methods presented their main innovations. Conversely, the first and third parts are common in both methods. The small displacement samples of the Tadcaster Bridge obtained from some satellite images of TerraSAR-X were applied to validate the proposed methods. The main conclusions of this study can be summarized as follows:

(1) In general, a long-term monitoring strategy contains the EOV conditions in measured data or features (i.e., displacement samples) extracted from raw measurements (i.e., SAR images). However, when there are inadequate or few data/feature samples, it is difficult to graphically observe their variations. The proposed strategy for augmenting the small data enabled us to better visualize the variations caused by the EOV conditions and any unknown variability sources.
(2) The proposed MCMC-ANN-MSD could handle the problem of the variability attributable to the environmental/operational conditions by obtaining smoother novelty scores. It was observed that the ANN based on the auto-associative neural network significantly reduced the EOV effects so that the results became unreliable without this tool.
(3) Due to some false alarms in the novelty scores of the training and validation samples, the MCMC-ANN-MSD method performed better via the EVT-based threshold estimator against the CLT.
(4) The proposed MCMC-TSL-MSD method better decreased the effects of the EOV conditions via obtaining smaller rates of false positive and false negative against the previous proposed method.
(5) Due to better performance of the MCMC-TSL-MSD method in terms of smaller rates of false positive and false negative, it was still successful in accurately making decisions via the classical CLT-based threshold estimator.

For further research, it is recommended to consider the possibilities of working more data such as cloud processing and data acquired from contact-based sensors, particularly for measuring EOV conditions. Moreover, it is suggested to compare the performances of the proposed methods with different sensing technologies and devices. Eventually, some important issues such as monitoring accuracy, sensitivity and detection range in SAR-based SHM technology should be investigated properly.
